# The distinct spatiotemporal distribution and effect of feed restriction on mtDNA copy number in broilers

**DOI:** 10.1038/s41598-020-60123-1

**Published:** 2020-02-24

**Authors:** Xiangli Zhang, Ting Wang, Jiefei Ji, Huanjie Wang, Xinghao Zhu, Pengfei Du, Yao Zhu, Yanqun Huang, Wen Chen

**Affiliations:** College of Livestock Husbandry and Veterinary Engineering, Henan Agricultural University, No. 15 Longzi Lake University Campus, Zhengzhou, 450046 P.R. China

**Keywords:** Animal breeding, Gene expression

## Abstract

Mitochondrial DNA (mtDNA) copy number reflects the abundance of mitochondria in cells and is dependent on the energy requirements of tissues. We hypothesized that the mtDNA copy number in poultry may change with age and tissue, and feed restriction may affect the growth and health of poultry by changing mtDNA content in a tissue-specific pattern. TaqMan real-time PCR was used to quantify mtDNA copy number using three different segments of the mitochondrial genome (*D-loop*, *ATP6*, and *ND6*) relative to the nuclear single-copy preproglucagon gene (*GCG*). The effect of sex, age, and dietary restriction (quantitative, energy, and protein restriction) on mtDNA copy number variation in the tissues of broilers was investigated. We found that mtDNA copy number varied among tissues (*P* < 0.01) and presented a distinct change in spatiotemporal pattern. After hatching, the number of mtDNA copies significantly decreased with age in the liver and increased in muscle tissues, including heart, pectoralis, and leg muscles. Newborn broilers (unfed) and embryos (E 11 and E 17) had similar mtDNA contents in muscle tissues. Among 42 d broilers, females had a higher mtDNA copy number than males in the tissues examined. Feed restriction (8–21 d) significantly reduced the body weight but did not significantly change the mtDNA copy number of 21 d broilers. After three weeks of compensatory growth (22–42 d), only the body weight of broilers with a quantitatively restricted diet remained significantly lower than that of broilers in the control group (*P* < 0.05), while any type of early feed restriction significantly reduced the mtDNA copy number in muscle tissues of 42 d broilers. In summary, the mtDNA copy number of broilers was regulated in a tissue- and age-specific manner. A similar pattern of spatiotemporal change in response to early feed restriction was found in the mtDNA content of muscle tissues, including cardiac and skeletal muscle, whereas liver mtDNA content changed differently with age and dietary restriction. It seems that early restrictions in feed could effectively lower the mtDNA content in muscle cells to reduce the tissue overload in broilers at 42 d to some degree.

## Introduction

Mitochondria are the only organelles in animal cells that possess their own independent genetic material, mitochondrial DNA (mtDNA), which is the control center of the life and death of a cell. In addition to energy production, mtDNA also plays an important role in metabolism, apoptosis, and intracellular signaling^1–3^. Mitochondria have a specialized genetic system that is responsible for the transcription and replication of mtDNA^4^. In contrast to the fixed (diploid) copy number of the nuclear genome, many copies of mtDNA exist within each cell, and these levels can fluctuate^5^. MtDNA copy number in mammals varies with age^6–8^, tissue^7,9^, and gender^10,11^. In human myocardial cells, there are approximately 6000 copies of mtDNA^9^, which is much higher than that the mtDNA copy number of skeletal muscle cells. Adipose tissue is the energy storage organ of the body, and its mtDNA copy number is maintained at a low number^12^. MtDNA copy number is a measure of mitochondrial function and reflects oxidant-induced cell damage^13^. The relative mtDNA copy number is approximately 3.7-fold higher in patients with non-alcoholic fatty liver disease than in healthy subjects^14^. Defective mtDNA replication can accelerate aging and reduce the lifespan of mice^15^. MtDNA copy number has been associated with various health issues. Deviations from the physiological number of mtDNA copies are expected to be deleterious and may contribute to aging^11^ and some inherited diseases, such as breast cancer^16^ and heart disease^17,18^, in humans. The mtDNA quantity in broilers was found to be correlated with the ascites phenotype in a tissue-specific manner^19,20^. Mitochondrial DNA content decreased significantly at the transformation phase in the spleen of a susceptible line but not in the line resistant to Marek’s Disease^21^. Heat stress reduced the mtDNA content in the liver of broilers, and feeding curcumin could prevent the decrease in mtDNA copy number to some degree^22^. Additionally, hepatic mtDNA copy number was not changed by dietary pyrroloquinoline quinone disodium in broilers^23^.

Caloric restriction delays the rate of aging in many species, such as yeast, worms, flies, and mice^24,25^. It was observed that caloric restriction could improve mitochondrial function in young nonobese adults by increasing the mtDNA content of skeletal muscle in association with a decrease in whole body oxygen consumption and DNA damage^25^. Feed restriction techniques, which are widely used in broiler production, can promote the balanced development of broilers in the early stages and reduce the incidence of leg disease and sudden death syndrome^26^. The weight loss caused by feeding restriction can be compensated by growth in the later stages^27^. The mechanism responsible for this is poorly understood.

Mitochondrial DNA copy number is a fundamental cellular bioenergetic phenotype. Based on the hypotheses that the mtDNA copy number in poultry may change with age and tissue, and feed restriction may affect poultry growth and health by changing the mtDNA content in a tissue-specific pattern, we investigated the shifts in the pattern of mtDNA copy number in tissues and the effects of different sexes, ages and feed restrictions on its variation by TaqMan fluorescence quantitative PCR technique (qPCR).

The ratio of mtDNA to nuclear DNA (mt/nucDNA) reflects the content of mitochondria (or mtDNA copy number) per cell in tissues. To improve accuracy, three genes located in different regions of the mtDNA genome were selected as marker genes^28^. The mean mtDNA copy number of each sample was measured based on the mt/nucDNA ratio of three mtDNA fragments: the *D-loop*, ATP synthase F0 subunit 6 (*ATP6)* and NADH dehydrogenase subunit VI (*ND6)*. The preproglucagon (*GCG*), a nuclear gene that is highly conserved among species and present as a single copy in animals, was used as the single-copy reference gene^29^.

## Results

### Variation in mtDNA copy number in different tissues

**Figure 1:**
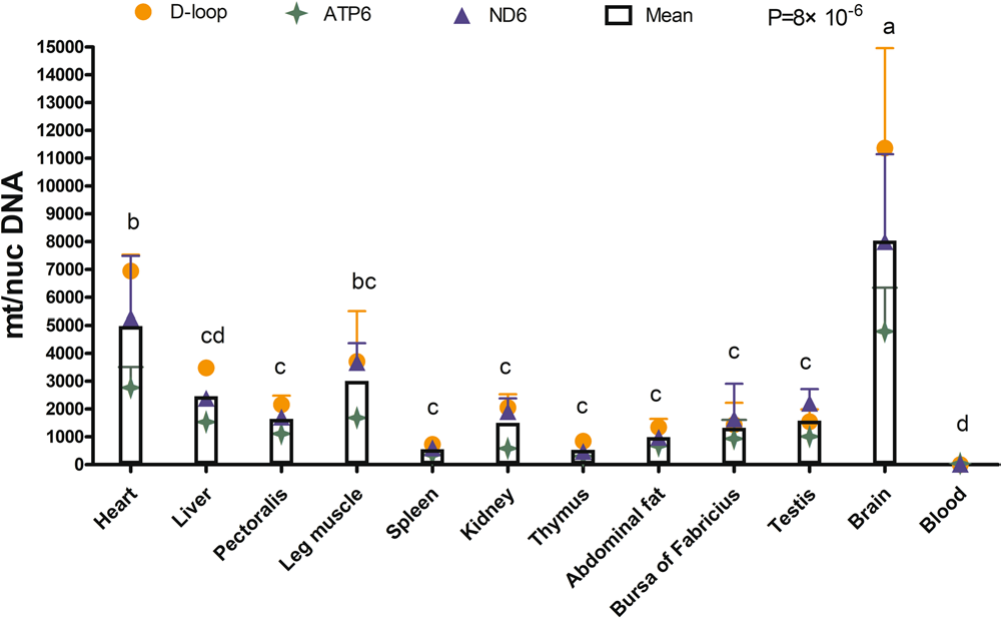
Variation in mtDNA copy number in various tissues of 21 d broilers. mt/nucDNA = mtDNA relative to nuclear DNA (*GCG*) copy number. Different letters indicate *P* < 0.05, and the same or no letter indicates *P* > 0.05. n = 3.

TaqMan qPCR was used to quantitatively analyze the variation in mtDNA copy number in 12 different tissues of 21 d male broilers with mitochondrial *D-loop*, *ND6*, and *ATP6* genes. First, we observed the pattern of change in tissues on the mt/nucDNA ratio of each individual mtDNA gene (*D-loop*/*ATP6*/*ND6*) (Supplementary Fig. S1, Table S1). The ratio of mt/nucDNA was the highest in most tissues for the *D-loop* gene and was the lowest in nearly all detected tissues for *ATP6*; however, the presented fluctuation patterns in tissues were similar for the three mtDNA genes, with the highest mtDNA content in the brain and the lowest mtDNA content in the blood (Supplementary Fig. S1). In addition, except in blood and liver tissues, there was no significant difference among the mt/nucDNA ratios of the three mtDNA genes in other tissues (Table S1). Therefore, the mean copy number based on three mtDNA genes was used for subsequent analysis. The results showed that there was a significant difference in the mean mtDNA copy number among the 12 different tissues of 21 d male broilers (*P* < 0.01, Fig. 1). The highest mtDNA copy number was found in brain cells (approximately 8040 copies/cell), followed by heart (approximately 4982 copies/cell), leg muscle, liver, pectoralis, testis, and other tissues, while blood had the smallest mtDNA copy number (less than ten copies). The number of mtDNA copies in the brain was higher than that in other tissues (*P* < 0.01), and the number of mtDNA copies in the heart was significantly higher than that in the liver and pectoralis (*P* < 0.05, Fig. 1).

### The mtDNA copy number in broilers at different developmental and growth stages

**Figure 2:**
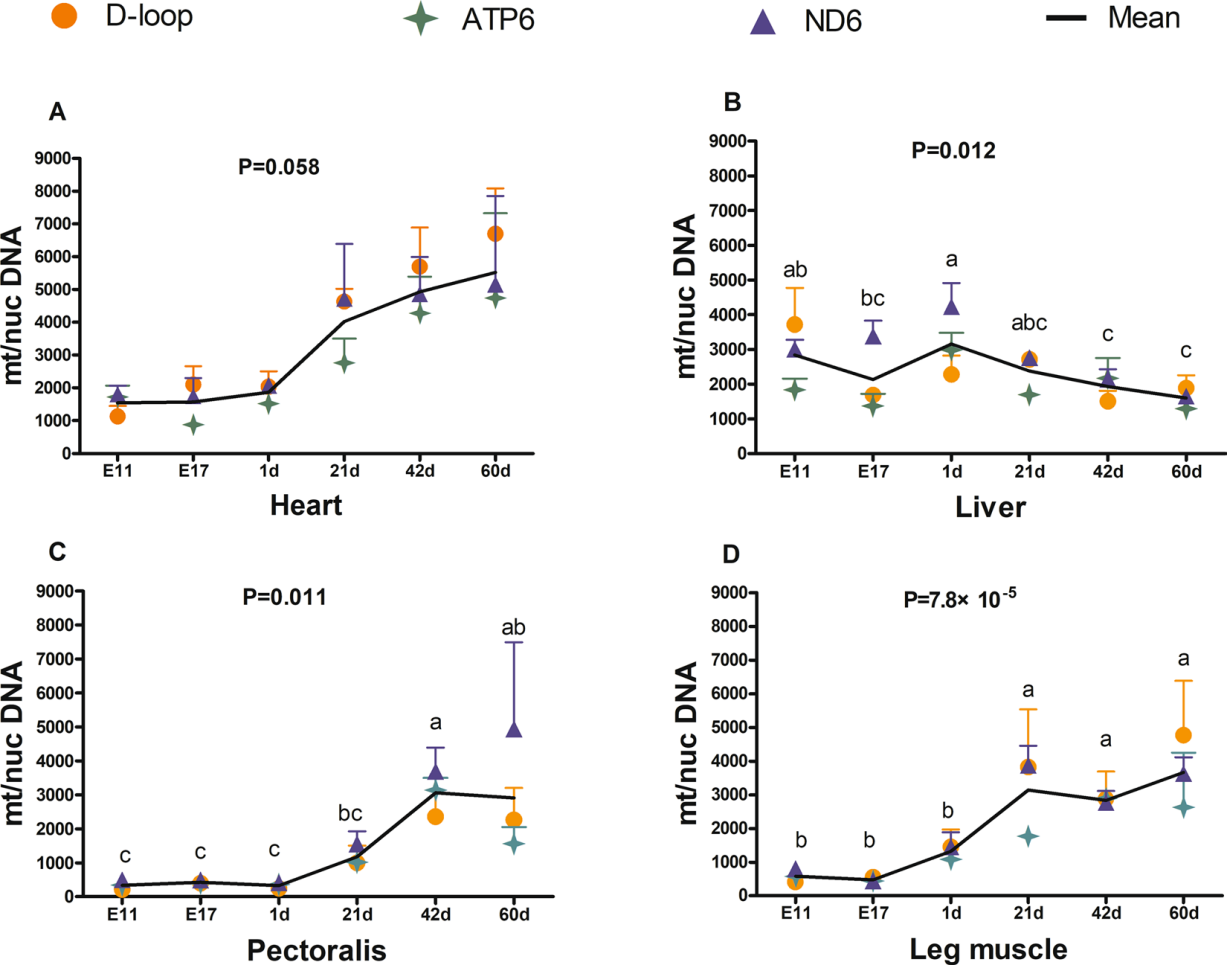
Change in mtDNA copy number with age in different tissues from broilers. (**A**) Heart; (**B**) liver; (**C**) pectoralis; (**D**) leg muscle. mt/nucDNA= mtDNA relative to nuclear DNA (*GCG*) copy number. Different letters indicate *P* < 0.05, and the same or no letter indicates *P* > 0.05. n = 3.

**Figure 3:**
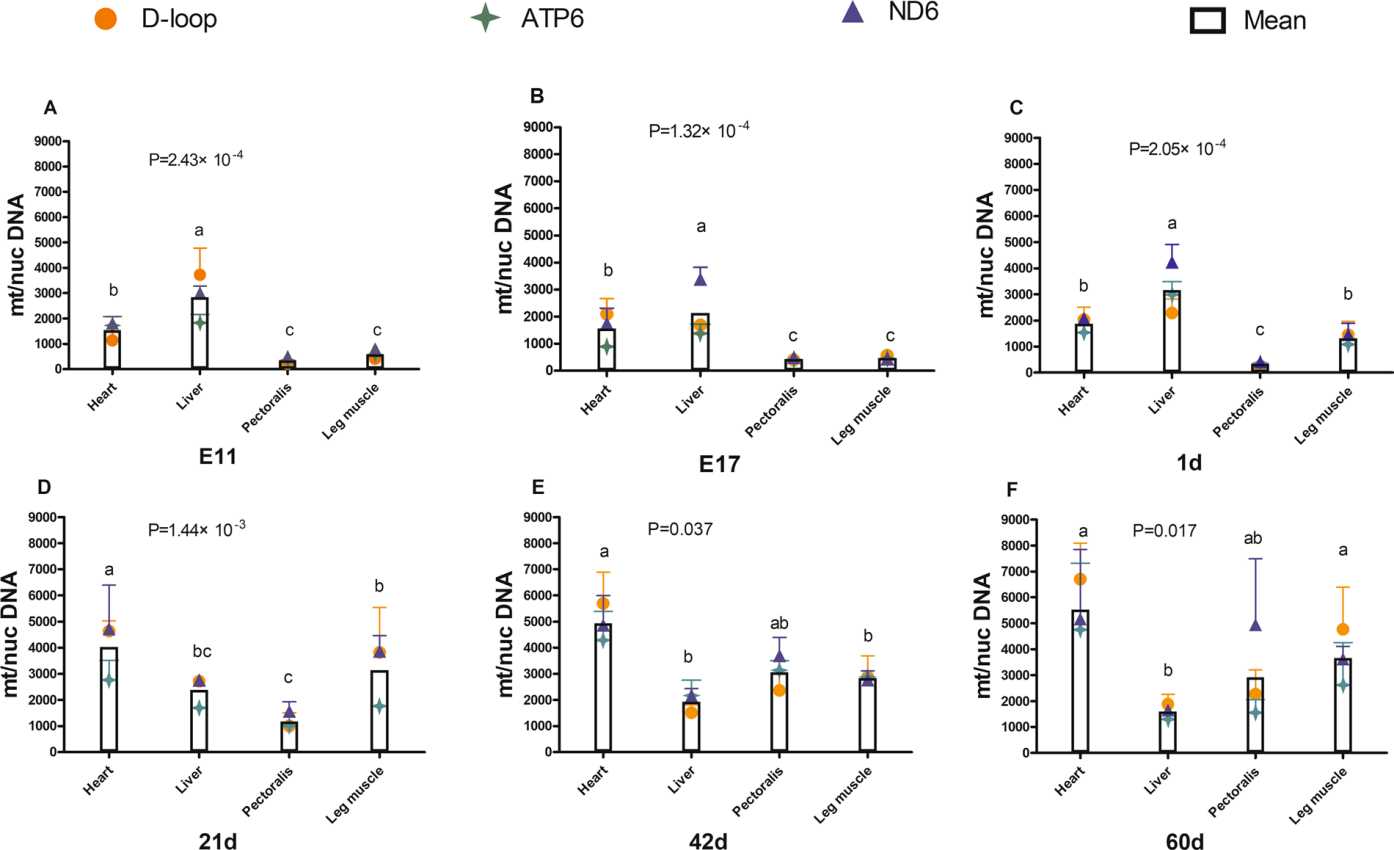
Variation in mtDNA copy number among tissues from broilers of different ages. (**A**) E11; (**B**) E17; (**C**) 1 d; (**D**) 21 d; (**E**) 42 d; (**F**) 60 d. mt/nucDNA = mtDNA relative to nuclear DNA (*GCG*) copy number. Different letters indicate significant differences (*P* < 0.05), and the same or no letter indicates *P* > 0.05. n = 3.

Based on the data from four tissues (heart, liver, pectoralis, and leg muscle) from broilers of different ages (E 11–60 d), two-way ANOVA was performed to analyze the effect of tissue and age on the mean mtDNA copy number. This analysis showed that the main effect of tissue (*P* = 7.94 × 10^−10^) and age (*P* = 1.20 × 10^−14^) on mtDNA copy number was significant, and their interaction on mtDNA copy number was also significant (*P* = 2.00 × 10^−8^). Therefore, we analyzed the effect of tissue and age on mtDNA copy number separately by one-way ANOVA (Figs. 2 and 3).

Overall, there was a similar change in the pattern of mean mtDNA copy number in the heart, pectoralis, and leg muscles with the development and growth of broilers, which maintained a low level at embryonic age (E 11 and E 17) and in newborn chicks (1 d), and then increased significantly with an increase in the age of chicks posthatching (Fig. 2A,C,D). The mtDNA copy number was significantly higher in broilers in the pectoralis at 42 d (Fig. 2C) and leg muscle at 21 d, 42 d, and 60 d (Fig. 2D), than that during the incubation period and at 1 d. However, the opposite trend occurred in the change in mtDNA copy number in the liver, which maintained a relatively high level before hatching and in newborn chicks and then significantly decreased with age (*P* < 0.05, Fig. 2B). The mtDNA copy number at 42 d and 60 d was significantly lower than that at E11 and 1 d in liver tissue (*P* < 0.05, Fig. 2B). In addition, there was no significant difference in mtDNA copy number between avian embryos (E 11 and E 17) and newborn chicks (1 d) in the heart, pectoralis and leg muscles (Fig. 2A,C,D), whereas the mtDNA copy number in the liver was significantly lower at E17 than in newly hatched chicks (Fig. 2B).

The mtDNA copy number presented clearly differential fluctuation patterns among tissues in different development and growth stages. At the embryonic stage (E11 and E17) and in newborn chicks, the mtDNA copy number follows the order: liver > heart > leg muscle > pectoralis; the mtDNA content in liver was significantly higher than that in other tissues; and the mtDNA content in heart was significantly higher than that in pectoralis (*P* < 0.01, Fig. 3A–C). After hatching, the mtDNA copy number was the highest in the heart of broilers from 21–60 d (*P* < 0.05, Fig. 3D–F) and was the lowest in the pectoralis at 21 d (Fig. 3D) and in the liver at 42 d (Fig. 3E) and 60 d (Fig. 3F). At 21 d, the mtDNA content in the heart was significantly higher than that in other tissues, and the mtDNA content in leg muscle was significantly higher than that in the pectoralis (Fig. 3D). At 42 d, the mtDNA content in the heart was significantly higher than that in the liver and leg muscle, and there was no significant difference among the leg muscle, pectoralis and liver (Fig. 3E). At 60 d, the mtDNA content in the heart and leg muscle was significantly higher than that in the liver, and no significant difference was observed among muscle tissues, including leg muscle, pectoralis, and heart (Fig. 3F).

### Effects of sex on mtDNA copy number in broilers

**Figure 4:**
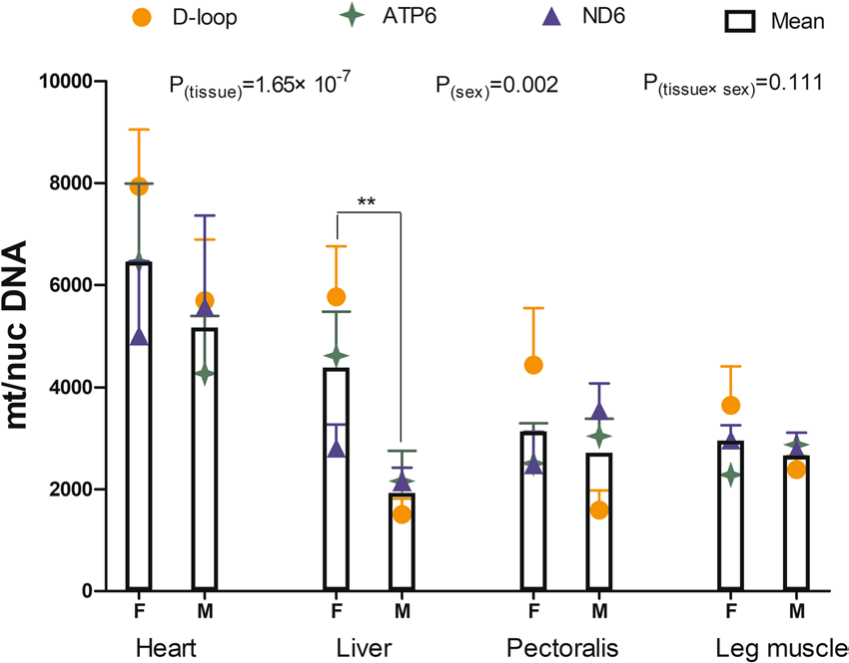
MtDNA copy number of male and female broilers. mt/nucDNA= mtDNA relative to nuclear DNA (*GCG*) copy number. The P values for sex and tissue determined by two-way ANOVA are presented in the figure. Comparisons between sexes in certain tissues were based on one-way ANOVA. **Indicates *P* ≤ 0.01, and no * indicates *P* > 0.01. Abbreviations: F, female; M, male. n = 3.

Based on the data from four tissues (heart, liver, pectoralis, and leg muscle) of 42 d female and male broilers (control group), two-way ANOVA was used to analyze the effect of tissue and sex. The interaction of tissue and sex was not significant (*P* = 0.111) on the mtDNA copy number of broilers at 42 d, whereas the main effect of sex (*P* = 0.002) and tissue (*P* = 1.65×10^−7^) was significant (Fig. 4). Overall, the mean number of mtDNA copies in female broilers was higher than that in male broilers (Fig. 4) and was approximately two times that in male broilers in the liver (*P* < 0.01).

### Effects of feed restriction on the growth and mtDNA copy number of 21 d broilers

After two weeks of restricted feeding (from 7–21 d), the body weight of 21 d broilers was significantly reduced. In addition, there were significant differences in body weight among the three feed restriction groups at 21 d, with protein restriction (PR) > energy restriction (ER) > quantitative restriction (QR). The growth of broilers in the QR group was seriously retarded: the body weight of the QR group was only 57.5% of that of the control group, and the liver weight, heart weight, pectoralis weight, and leg muscle weight of the QR group were significantly lower than those of other groups (*P* < 0.05). In addition, the weights of the liver and pectoralis in the ER group were significantly lower than those in the control group (Table 1).

**Table 1:** Effects of feed restriction on the body weight and tissue weight of broilers (unit: g).

Age	Group^a^	Body weight^b^	Heart weight^c^	Liver weight^c^	Pectoralis weight^c^	Leg muscle weight^c^
21 d	Control	824.13 ± 11.55^a^	5.37 ± 0.21^ab^	20.93 ± 0.63^b^	147.23 ± 4.40^a^	102.28 ± 3.84^a^
	QR	474.13 ± 6.80^d^	2.81 ± 0.05^c^	11.49 ± 0.45^d^	65.31 ± 2.21^c^	57.80 ± 1.74^b^
	ER	730.50 ± 685^c^	4.69 ± 0.24^ab^	16.56 ± 0.88^c^	131.85 ± 4.18^b^	92.37 ± 3.04^a^
	PR	789.13 ± 7.16^b^	7.20 ± 1.30^a^	25.03 ± 2.19^a^	138.10 ± 4.91^ab^	100.07 ± 4.15^a^
42 d	Control	2519.25 ± 67.27^a^	9.54 ± 0.67	49.51 ± 3.70	525.60 ± 2.86	384.45 ± 14.62
	QR	2318.50 ± 69.38^b^	9.75 ± 0.63	44.30 ± 2.59	455.37 ± 16.00	357.22 ± 13.43
	ER	2371.75 ± 53.29^ab^	8.99 ± 0.88	42.94 ± 2.02	492.85 ± 29.07	356.35 ± 15.41
	PR	2341.12 ± 56.45^ab^	10.24 ± 0.48	49.00 ± 1.82	502.62 ± 25.59	362.90 ± 13.57

Note: ^a-d^different lowercase letters in different groups of the same age indicate *P* < 0.05.

^a^In 8–21 d, the control group was fed a conventional diet ad libitum, the QR group was fed a conventional diet between 8:00–13:00, the ER group was fed a 15% energy-restricted diet, and the PR group was fed a 15% protein-limited diet. At 22–42 d, the four groups of broilers were transferred to the same conventional diet ad libitum.

^b^Body weight refers to the average live weight of each group of broilers (n = 20).

^c^Tissue weight refers to the tissue weight of the slaughtered broilers (n = 3).

**Figure 5:**
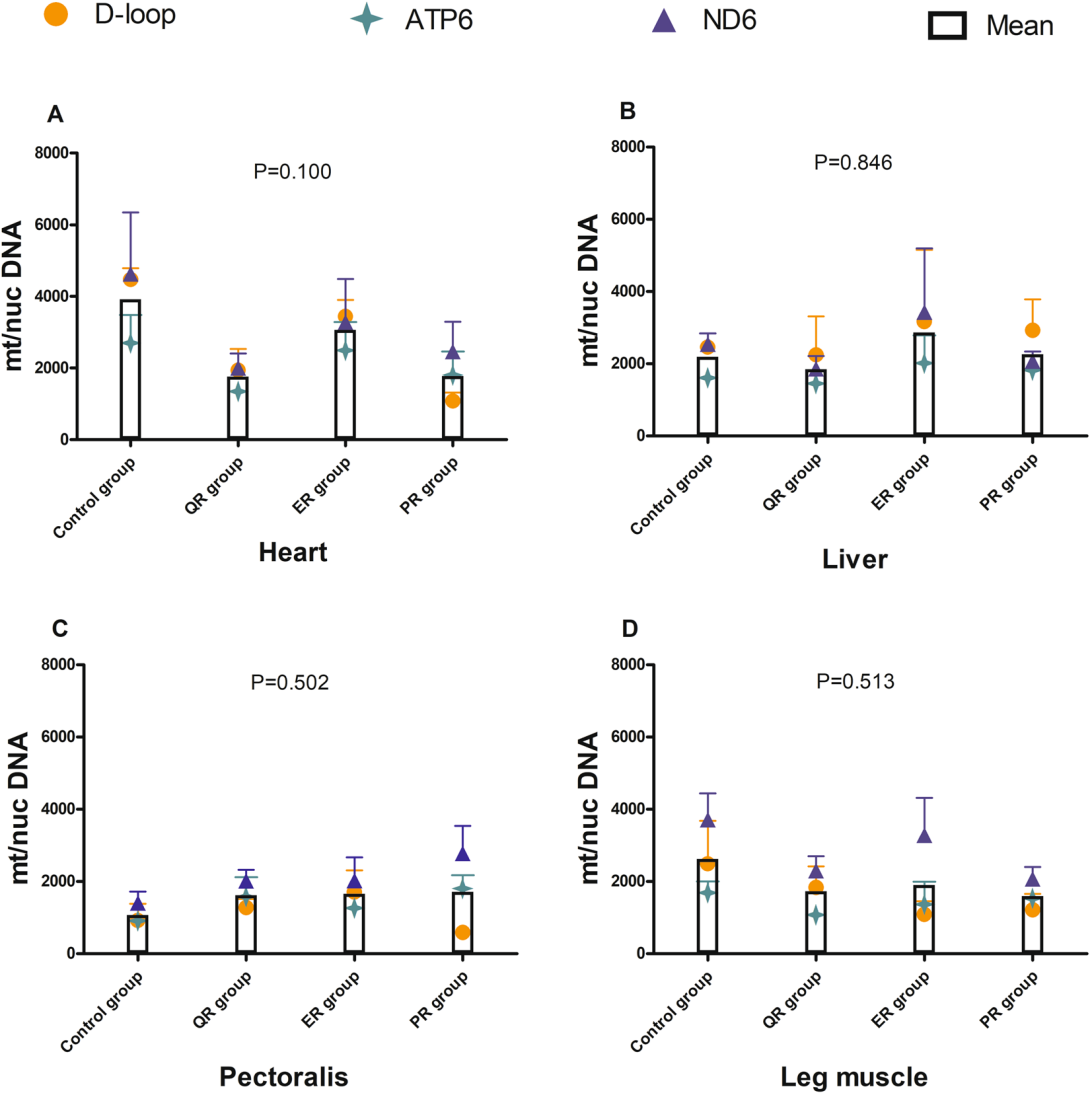
Effects of feed restriction on the mtDNA copy number of 21 d broilers in different tissues. (**A**) Heart; (**B**) liver; (**C**) pectoralis; (**D**) leg muscle. *P*_group_ = 0.229; mt/nucDNA= mtDNA relative to nuclear DNA (*GCG*) copy number. *P*_group_ × _tissue_ = 0.453. The control group was fed a conventional diet ad libitum, the QR group was fed a conventional diet between 8:00–13:00 from 8–21 d, the ER group was fed a 15% energy-restricted diet ad libitum from 8–21 d, and the PR group was fed a 15% protein-limited diet ad libitum from 8–21 d. n = 3.

Further analysis by one-way ANOVA revealed that feed restriction (*P* > 0.05) for two weeks did not significantly change the mtDNA content of 21 d broilers in four different tissues, including the heart, liver, pectoralis, and leg muscles (Fig. 5). There was no significant difference in mtDNA copy number among the four groups in any detected tissue (Fig. 5). The copy number of the three restriction groups in heart and leg muscle tissues was slightly lower than that in the control group (*P* > 0.05).

### Effects of feed restriction on the growth and mtDNA copy number of 42 d broilers

After resuming a conventional diet ad libitum at 22 d, there was a strong compensation in the growth of broilers in the three feed restriction groups (Table 1). At 42 d, the body weight of the ER group and PR group reached 94.12% and 92.93% of that of the control group, respectively, and only the body weight of the QR group was significantly lower than that of the control group (*P* < 0.05). There was no significant difference among the body weights of the three restricted-feeding groups (*P* > 0.05). In addition, the weights of the heart, liver, pectoralis, and leg muscles were not significantly different among the four groups (Table 1). In the QR group, the pectoralis weight was 86.5% of that of the control group, and the leg muscle weight was 92.9% of that of the control group.

**Figure 6:**
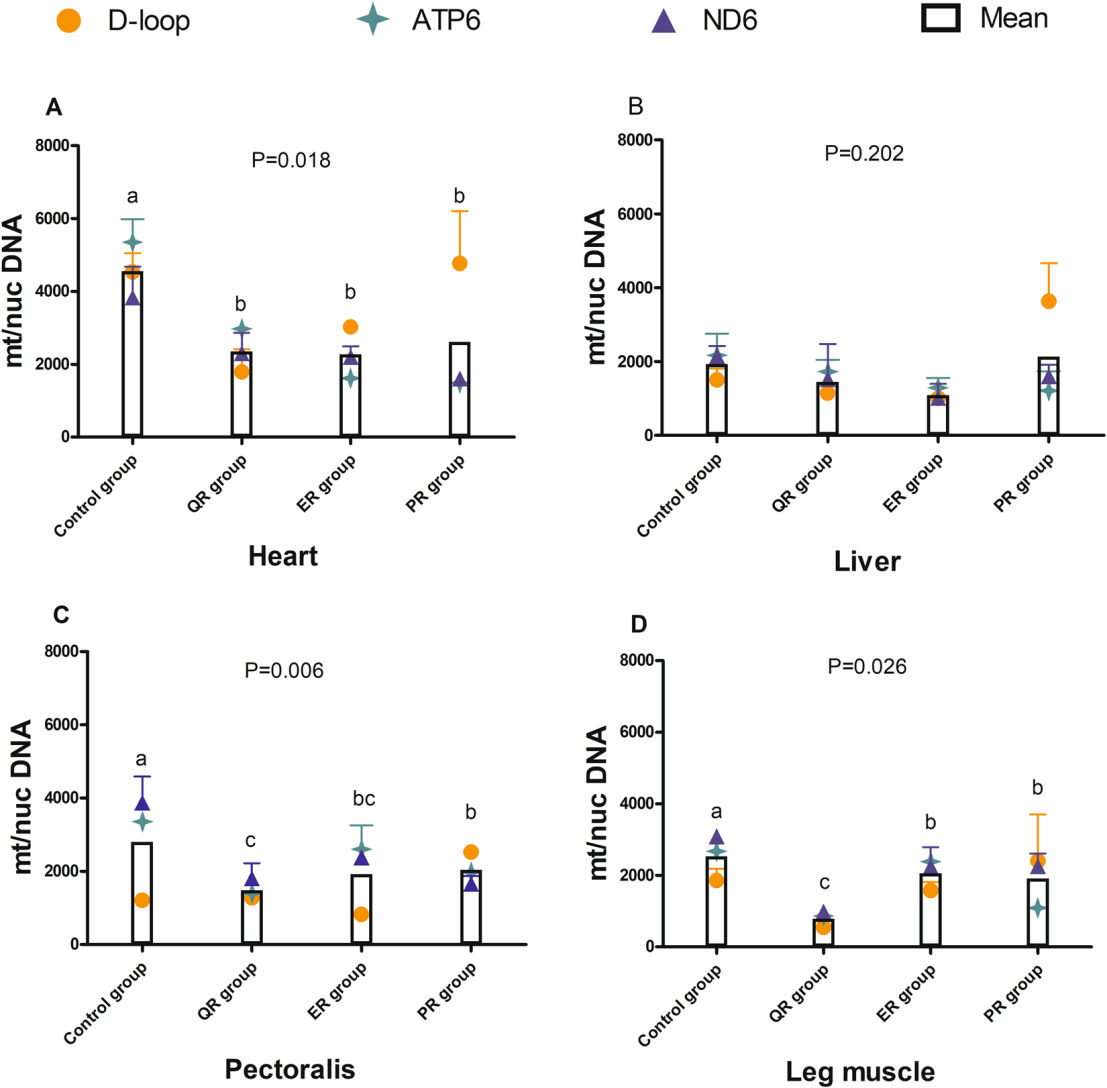
Effects of feed restriction on the mtDNA copy number of 42 d broilers in different tissues. (**A**) Heart; (**B**) liver; (**C**) pectoralis; (**D**) leg muscle. mt/nucDNA= mtDNA relative to nuclear DNA (*GCG*) copy number. *P*_group_ = 0.002; *P*_group_ × _tissue_ = 0.472. Different letters indicate *P* < 0.05, and the same or no letter indicates *P* > 0.05. The control group was fed a conventional diet ad libitum, the QR group was fed a conventional diet between 8:00–13:00 from 8–21 d, the ER group was fed a 15% energy-restricted diet ad libitum from 8–21 d, and the PR group was fed a 15% protein limited diet ad libitum from 8–21 d. n = 3.

Early feed restriction (8–21 d) significantly reduced the mtDNA copy number in the heart (*P* = 0.018, Fig. 6A), pectoralis (*P* = 0.006, Fig. 6C), and leg muscle (*P* = 0.026, Fig. 6D) of 42 d broilers. The interaction of the feed restriction method and tissue on mtDNA copy number was not significant (*P* = 0.472) at 42 d. The mtDNA content of the control group was significantly higher than that of the QR, ER and PR groups in heart, pectoralis and leg muscle (*P* < 0.05). In addition, the mtDNA copy number of the QR group was significantly lower than that of the PR group in the pectoralis (Fig. 6C) and that of the PR and ER groups in leg muscles (Fig. 6D). However, no differences were observed in the heart among the three restriction groups (Fig. 6A) and in the liver among the four groups (Fig. 6B).

## Discussion

Mitochondrial biogenesis is stimulated in response to increased energy demand and is hallmarked by a characteristic increase in cellular mtDNA level^30^. According to the energy demand and oxygen consumption of cells^31^, the mtDNA copy number varies among tissues and changes with growth and development.

Al-Zahrani *et al*. observed an increase in mtDNA copy number with age (from 3 weeks to 20 weeks) in the breast muscle of male broilers from both ascites-resistant lines and the unselected population but not in the ascites-susceptible line^20^. Our research indicated that the mtDNA copy number in broilers was regulated in a tissue-related manner with age. We observed a similar temporal shift in mtDNA copy number in the muscle tissues of birds, including the heart, pectoralis and leg muscle. However, changes in mtDNA copy number in the liver presented an opposite trend to that in muscle tissues with age, which resulted in a distinct spatial fluctuation in the pattern of mtDNA copy number in tissues during the embryonic development stage and after hatching. In addition, we also observed that mtDNA content declined in muscle tissues, including the heart, pectoralis and leg muscle tissues, of 42 d broilers but not in the liver after early dietary restriction.

The increase in mtDNA copy number in muscle tissues with age after hatching aligns with the requirement of cellular energy metabolism during the development and growth stages of broilers. After birth, broilers rapidly grow (especially their skeletal muscles), and their hearts and skeletal muscles are required to provide additional energy by increasing mtDNA content and mitochondrial biogenesis.

The liver is the main site of fat metabolism in poultry, and some studies have shown that 90% of the energy needed for the development of chicken embryos comes from lipids in the yolk^32^. Only a small amount of glucose is used for lipogenesis (fatty acid synthesis) in the liver of avian embryos; this can increase to a substantial plateau level within 8 d after hatching due to feeding^33,34^. Glycogen concentrations in the liver of birds increased threefold from E11 to a maximum at day 18^35^. After hatching, the number of mtDNA copies decreased gradually in the liver with an increase in age. This may be because the energy needed by the body after hatching comes not only from lipid metabolism but also from glucose and protein metabolism.

Several attempts have been made to clarify the relationship between mtDNA copy number and age in mammals, but there is currently much uncertainty^6,7,36–39^. It was reported that there was a dramatic increase in the mtDNA copy number of the human heart one year after birth^37^. Wachsmuth *et al*. found that human male skeletal muscle showed an age-related (3–96 years) decrease in mtDNA copy number, while there was an age-related increase in mtDNA copy number in the liver^7^. Miller *et al*. did not find a significant change in mtDNA copy number with age in skeletal muscle and myocardium^9^. Frahm *et al*. did not observe a significant change in mtDNA copy number in skeletal muscle, heart, and other tissues in humans from two months to 93 years of age^38^. In addition, it was observed that mtDNA copy number increased significantly in pig liver from birth to 180 d and significantly decreased from 180 d to 7 years^39^. The mtDNA content in mice increased gradually with age in the heart, lung, kidney, spleen, and skeletal muscle, whereas the amount of mtDNA in the liver increased steadily until 5 months of age^6^.

The difference in the mtDNA copy number among tissues reflects the intensity of normal energy metabolism in different tissues in the organism^6^. The number of mitochondria and the mtDNA copy number in different types of cells depends on their energy requirements and is related to the metabolic activity of the cells in the tissue^40^. As in humans^7^, mice^6^, and pigs^39^, the mtDNA content of broilers shifts dramatically in different tissues, with a high number of copies in the brain, heart, and skeletal muscles and a low number of copies in peripheral blood. In accordance with our results (Fig. 1), Reverter *et al*. reported that the relative mtDNA content in tissues was heart > leg muscle > breast muscle > fat in broilers at 48 d^41^. In the present study, we confirmed that the sex-specific regulation of mtDNA levels occurs in poultry, as it does in humans^11^ and mice^10^. Al-Zahrani *et al*. found that tissue and sex differences in mtDNA content varied between different lines with ascites resistance/susceptibility^20^. Female broilers had a higher mtDNA copy number than males in the ascites-resistant line, while male broilers had a higher copy number than females in the ascites-susceptible line^20^. Previous experiments found that female rats were less prone to mtDNA injury by reactive oxygen species^38^.

The genetic selection for a high muscle to bone ratio and the high calorie content of a ration result in tissue overload in broilers, which causes significant mortality from cardiovascular disease such as sudden death syndrome and ascites^42^. In addition, rapid growth can induce severe lameness, bone defects, and deformity in broilers^42^. Ascites appears to be mainly caused by the high metabolic oxygen requirement of rapid growth combined with the insufficient capacity of the pulmonary capillaries^43–45^. Early feed restriction could reduce the incidence of ascites and the mortality of broilers^46,47^. It was found that male birds of a ascites-susceptible line had a significantly higher mtDNA copy number in the breast muscle than male birds of the resistant line at both 3 weeks and 22 weeks^19,20^. We observed that early feed restriction (by any method) could effectively reduce the mtDNA copy number in the muscle tissues, including heart, pectoralis, and leg muscles, of 42 d broilers (but not 21 d broilers), which indicates that feed restriction reduces the mtDNA copy number in the muscle tissues of broilers in a time-dependent manner. It seems that feed restriction may improve the health of fast-growing broilers (including a reduction in the incidence of ascites and mortality) through reducing the mtDNA copy number to lower the overload of muscle tissues such as skeletal tissue and heart to some degree. The liver tissue of broilers does not seem to be sensitive to early feed restriction. It was reported that the maternal protein diet decreased hepatic mtDNA copy number in male newborn piglets by affecting the epigenetic regulation of hepatic mtDNA transcription^48^. Caloric restriction in a 6-month intervention increased mtDNA content in the skeletal muscle of young nonobese adults^25^. The opposite effect of caloric restriction on mtDNA copy number in skeletal muscle between humans and broilers may be related to the difference in the population (nonobese or obese phenotypes).

Dietary changes have a pronounced effect on the tissue metabolic strategy and mitochondrial phenotypes^49–52^. In response to changes in energy demand and supply, the organism regulates mitochondrial metabolic status to coordinate ATP production^49,50^. It has been shown that caloric restriction reduces the generation of free radicals by mitochondria in parallel to a reduction in mitochondrial proton leaks^51,52^.

## Conclusion

The mtDNA copy number of broilers dramatically fluctuated among tissues and was regulated in an age- and tissue-specific manner. The mtDNA copy number in female broilers was higher than that in male broilers. Newborn broilers and E 11 and E 17 embryos had a similar level of mtDNA content in the muscle tissues assessed. The number of mtDNA copies significantly increased with age after hatching in the muscle tissues, including the heart, pectoralis, and leg muscle tissues. Different approaches to early feed restriction effectively lowered the mtDNA content in muscle tissues of 42 d broilers. In contrast to muscle tissues, the liver presented an opposite temporal pattern, with the mtDNA copy number declining after hatching, and the mtDNA copy number in the liver was not sensitive to early feed restriction in 42 d broilers.

## Materials and Methods

### Animals

A total of 120 newborn (1 d) avian broilers were cage-raised. Feed (with conventional diet) and water were available ad libitum with 23 h illumination. The room temperature was maintained at 33 °C from 0–3 d, 30 °C from 4–7 d, 27 °C from 8–21 d, and 24 °C from 22–42 d. Bird management was consistent with the recommendations of the AA Broiler Management Guide^53^. At the age of 7 d, 80 broilers with similar weights were selected from the population and cage-raised separately and randomly divided into four experimental groups (n = 20/group, male: female = 1:1): control group, QR group, ER group and PR group. From 8–21 d, the control group was fed a conventional diet ad libitum, the QR group was only provided a conventional diet between 8:00–13:00, the ER group was fed a diet with 15% energy limitation (energy-restricted diet) ad libitum, and the PR group was fed a 15% protein-limited diet (protein-restricted diet) ad libitum. From 22–42 d, the four groups of broilers were transferred to the same conventional diet ad libitum. The composition and nutrient levels of the experimental diets are presented in Supplementary Table S2. The diet was prepared according to the nutritional requirements of broilers recommended by NY/T 33-(2004) in China. At 21 d and 42 d, the broilers of each group were weighed.

### Sample collection

Broilers (close to the average weight of the same sex) were selected and sacrificed at 1 d (newborn chicks, no feeding, n = 3, male broilers), 21 d (n = 3 for each group of male control and QR, ER, and PR broilers), 42 d (n = 3 for each group of female and male control broilers; n = 3 for each group of male QR, ER, and PR broilers) and 60 d (n = 3 for male control broilers). The following tissues were collected: brain, liver, heart, spleen, kidney, abdominal fat, thymus, bursa of Fabricius, testis, pectoralis, leg muscle, and blood. Broilers were fasted for 12 h before slaughter. In addition, heart, liver, pectoralis, and leg muscles were collected from broiler embryos at embryonic ages 11 (E 11, n = 3) and 17 (E 17, n = 3). The collected tissue samples were washed with normal saline, snap-frozen with liquid nitrogen and stored at −80 °C until DNA extraction. All procedures were approved by the Animal Care and Use Committee of Henan Agricultural University (Zhengzhou, China).

### DNA extraction

Total DNA was extracted using an animal tissue DNA extraction kit (LifeFeng, Shanghai, China). The concentration and purity of DNA were determined by 1% agarose gel electrophoresis and a NanoDrop 2000 spectrophotometer (Thermo Scientific, Wilmington, DE, USA). DNA was diluted to 100 ng/μL and deposited at −20 °C for use.

### Primer design and standard preparation for TaqMan qPCR

The ratio of mtDNA to nuclear DNA reflects the content of mitochondria per cell in tissues. To reduce the error, the mtDNA copy number of each sample was measured based on the mean copy number of three mtDNA fragments relative to a single-copy nuclear gene. The primers and probes for the three mtDNA genes, namely, *D-loop*, *ATP6*, and *ND6* (accession no: X52392.1), and nuclear gene *GCG* (accession no: DQ185929.1) were optimized and are presented in Table 2. In the primer design for the mtDNA genes, potential interferences, such as nuclear pseudogenes and reported high frequency fragments of insertion/deletion, were avoided through online NCBI BLAST (https://www.ncbi.nlm.nih.gov/blast), and finally, the primers and probes were synthesized by Bioengineering (Shanghai) Co., Ltd. (HAP purification).

**Table 2:** The primers and probes used for qPCR.

Gene Name	Primer and Probe Sequence (5′ to 3′)	Amplicon Size (bp)	Annealing Temperature (°C)
*D-loop*	F: 5′ACCCCTGCCTGTAATGTACTTC3′ R: 5′CACGGACTAAAGAGGGGAAGAT3′ Probe: 5′TTCTTTCCCCCTACACCCCTCGCCCT3′	183	60
*ATP6*	F: 5′ATTCTCAAGCCCCTGCCTAC3′ R: 5′TCAGAGTTGGATGGTGGAGAGG3′ Probe: 5′CCTCCCATCACTCCTTCTTCCAGCCCTC3′	124	60
*ND6*	F: 5′TAACAACAAACCTCACCCAGCC3′ R: 5′GTGTGTCTTTTGCTCGGTTGGA3′ Probe: 5′AGCCACCAAAAACAACCCAACCCCAC3′	95	60
*GCG*	F: 5′GTGGAGGGCTGATAAAACACAAT3′ R: 5′TCCAACTCCTTGACCTCTATCC3′ Probe: 5′TTCAGCCCTCAGCATTCAGTCCCATT3′	205	60

The mitochondrial genes and nuclear genes were amplified with the corresponding primers, and the PCR products were recovered and purified by a Tiangen gel recovery kit (Tiangen, Beijing, China) and subcloned into a pMD18-T vector (Takara, Dalian, China). The positive plasmids verified by PCR were extracted by a column plasmid extraction kit (Takara, Dalian, China) and confirmed by sequencing. The plasmid concentration was measured by a spectrophotometer, and the plasmid copy number was calculated as follows: plasmid concentration (ng/μL) = OD_260_ value × 50 mL × dilution final volume (mL) × 1000/diluted original solution volume (μL), and plasmid copy number (copy/μL) = plasmid concentration (ng/μL) × 6.02 × 10^14^/(DNA length × 660)^54^.

The templates for the qPCR standard curve were obtained by continuously diluting plasmids containing the target PCR product to 10^3^–10^10^ copies/μL.

### Quantitation of mitochondrial and nuclear genes

The double standard curve method was used to quantify the mtDNA and nuclear genes by Bio-Rad CFX96 (Bio-Rad Laboratories, Hercules, CA, USA). The standard curve was created by analyzing serial dilutions of plasmid DNA. The qPCR reaction was conducted in a total volume of 10 μL, which included 5.0 μL 2 × GoldStar TaqMan Mixture, 0.7 μL probe, 0.5 μL upstream primer, 0.5 μL downstream primer, 0.5 μL DNA templates (approximately 50 ng), and 2.8 μL deionized water, and under the following conditions: initial denaturation at 95 °C for 5 min; 40 cycles of 95 °C for 10 s, 60 °C for 30 s, and 72 °C for 30 s; and 72 °C for 1 min. PCR assays were performed in triplicate for each sample.

### Statistical analysis

First, mtDNA copy number was measured based on the qPCR data of each mtDNA gene. Considering that nuclear genes are diploid, mtDNA copy number (measured by certain mtDNA gene) = 2 × the copy number of *D-loop* (or *ATP6* or *ND6*)/*GCG* copy number. The mean mtDNA copy number of three mtDNA genes was used to conduct the related statistical analysis. Data were analyzed by SPSS 24.0, and the results are expressed as the mean ± standard error. Two-way ANOVA was used to analyze the association between tissue and age, sex and feed restriction on mtDNA copy number. If the interaction was significant, the data were analyzed again by one-way ANOVA. Significant differences in the data were identified by Duncan’s multiple range tests. The level of statistical significance was set as *P* < 0.05.

### Ethics approval and consent to participate

All animal procedures were conducted in accordance with the Guide for the Care and Use of Laboratory Animals and were approved by the Animal Care and Use Committee of Henan Agricultural University.

## Supplementary information

Supplementary information.

## Data Availability

All the data presented in the manuscript are available to readers.
