# Supplementary material for: The distinct spatiotemporal distribution and effect of feed restriction on mtDNA copy number in broilers
Source: Sci Rep. 2020 Feb 24;10:3240. doi: 10.1038/s41598-020-60123-1 (PMC7039872; doi:10.1038/s41598-020-60123-1)
Supplement: Supplementary file 1 — Supplementary information. [file 41598_2020_60123_MOESM1_ESM.pdf]

The distinct spatiotemporal distribution and effect of feed restriction on mtDNA copy number in broilers

Xiangli Zhang, Ting Wang, Jiefei Ji, Huanjie Wang, Xinghao Zhu, Pengfei Du, Yao Zhu, Yanqun Huang* & Wen Chen

College of Livestock Husbandry and Veterinary Engineering, Henan Agricultural University, No. 15 Longzi Lake University Campus, Zhengzhou, 450046, P. R. China.

*email: hyanqun@aliyun.com

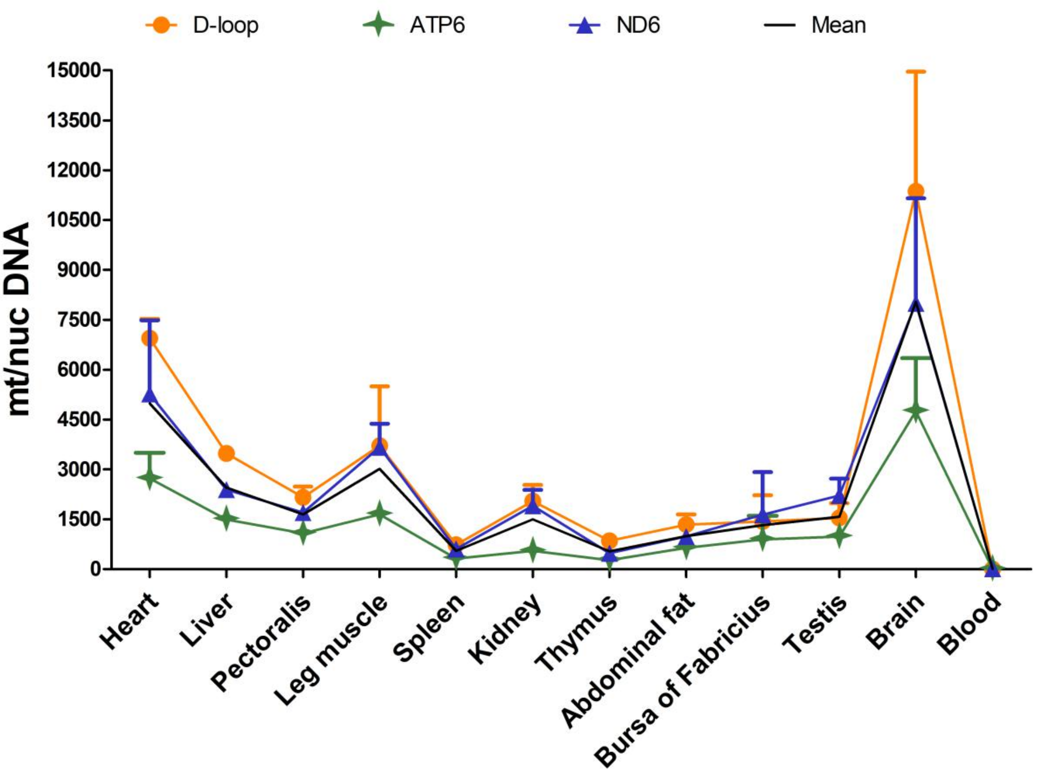

Figure S1 Variation of *D-loop*, *AT6*, and *ND6* in various tissues of 21 d broilers. mt/ nucDNA= mtDNA relative to nuclear DNA (*GCG*) copy number. n=3.

Table S1 Variation of *D-loop*, *AT6*, and *ND6* in various tissues of 21 d broilers

Tissue/Gene	D-loop	ATP6	ND6	Mean
Heart	6946.32±586.31	2730.54±777.58	5269.28±2221.82	4982.05
Liver	3485.59±104.35[a]	1498.36±96.78[c]	2389.74±209.36[b]	2457.90
Pectoralis	2160.59±318.99	1072.90±123.09	1708.53±175.49	1647.34
Leg muscle	3713.92±1787.81	1648.17±346.05	3673.45±697.22	3011.85
Spleen	728.58±225.40	318.43±52.91	605.47±43.17	550.83
Kidney	2051.46±483.03	548.91±58.69	1910.40±471.98	1503.59
Thymus	849.88±239.28	269.22±148.93	477.63±100.20	532.24
Abdominal Fat	1343.36±303.47	639.23±65.53	980.78±22.57	987.79
Bursa of Fabricius	1436.85±788.87	893.65±713.75	1643.65±1270.44	1324.72
Testis	1544.32±442.60	978.55±241.17	2213.25±507.08	1578.71
Brain	11366.0±3597.27	4751.19±1598.45	8002.96±3146.76	8040.05
Blood	5.32±1.23[b]	6.34±0.45[b]	16.58±2.98[a]	9.41

Note: [a, b] different lowercase letters in the same row indicate significant difference ($P < 0.05$).

Table S2 Composition and nutrient levels of experimental diets (air-dry basis) %

Items	1-21 d			22-42 d
	Conventional diet	Energy restriction diet	Protein restriction diet	Conventional diet
Ingredients				
Corn	45.25	44.29	53.66	49.64
Corn germ meal	5.20	16.00	9.00	8.00
Soybean oil	6.50	0.40	5.60	6.00
Soybean meal(CP 43%)	36.45	33.43	24.62	30.65
Fish meal(CP 60%)	2.00	1.00	2.00	1.50
$CaCO_3$	1.26	1.27	1.30	1.19
$CaHPO_4$	1.62	1.80	1.71	1.43
Choline chloride	0.26	0.26	0.26	0.20
Lys		0.07	0.34	
Met	0.16	0.18	0.21	0.09
NaCl	0.3	0.30	0.30	0.30
Premix[1)]	1.00	1.00	1.00	1.00
Total	100.00	100.00	100.00	100.00
Nutrient levels				
ME/(MJ/kg)	12.97	11.05	12.97	12.97
CP	22.23	22.28	18.76	20.12
Ca	1.02	1.04	1.02	0.91
TP	0.73	0.85	0.74	0.70
NPP	0.45	0.45	0.45	0.40
AP	0.44	0.45	0.44	0.41
Lys	1.22	1.22	1.22	1.07
Met	0.50	0.50	0.50	0.40
Cys	0.35	0.36	0.30	0.33

[1)] The premix provided the following per kg of diets: VA 2700IU, VD 3400IU, VE 10IU, VK 0.5mg, VB_1 2.0mg, VB_2 5mg, VB6 3.0mg, VB12 0.007mg, nicotinic acid 30mg, pantothenic acid 10mg, folic acid 0.50mg, biotin 0.10mg, chloride 750mg, Cu 8mg, Zn 80mg, Fe 80mg, Mn 80mg, Se 0.30mg, I 0.7mg.

CP: crude protein; Ca: calcium; TP: total phosphorus; NPP: non-phytate phosphorus; AP: available phosphorus.
